# Enhancing the Reliability of Shearing Tools: A Modular Approach with Weld Deposition Technology

**DOI:** 10.3390/ma18071527

**Published:** 2025-03-28

**Authors:** Daniela Maria Iovanas, Adela-Eliza Dumitrascu

**Affiliations:** 1 Department of Materials Engineering and Welding, Transilvania University of Brasov, 1 Colina Universitatii, 500036 Brasov, Romania; daniela.iovanas@unitbv.ro; 2 Department of Manufacturing Engineering, Transilvania University of Brasov, 5 Mihai Viteazul, 500036 Brasov, Romania

**Keywords:** reliability analysis, shearing tools, interchangeable modulated elements, welding deposition, shearing tool performance

## Abstract

The increasing demand for sustainable and cost-effective manufacturing solutions has led to the development of innovative approaches to enhance the durability and reliability of cutting tools. This study presents a novel method for manufacturing shearing tools utilizing interchangeable modular elements loaded by deposition welding with covered electrodes. Using Weibull distribution modeling, a comparative reliability analysis between conventionally manufactured shear tools and the proposed modular design demonstrates a significant increase in the mean time to failure (MTTF). The least squares method (LSM) estimation was used in order to determine the shearing tools’ lifetime, expressed by reliability indices. Experimental results confirm that the modular tools achieve more than double the lifetime of traditional counterparts, with improved resistance to wear and mechanical stress. These findings highlight the potential for widespread industrial application, optimizing tool performance and sustainability in manufacturing processes.

## 1. Introduction

The development of global industry in recent decades has led to increasingly intensive exploitation of resources. This has resulted in shortages of raw materials, resources, and energy. In order to counteract this, it has been necessary to find solutions for the realization of products based on the principles of sustainable development, reliability, availability, and increased productivity; these solutions include reducing repair times, manufacturing costs, and maintenance [1].

Various materials in the form of blanks, sheets, bars, rods, pipes, profiles, etc., are used to produce finished products, which are cut to smaller sizes needed for further processing.

Currently, cutting processes are widely used in industry, including laser cutting [2,3], plasma cutting [3,4], and other conventional cutting methods, but they often require expensive installations and high energy consumption. Mechanical shear cutting is a commonly used cutting process, due to its advantages such as low cost, high cutting speed, high productivity, affordable equipment, and ease of operation [3].

The analysis of used tools in mechanical shear cutting has been a relevant topic for many researchers. Consequently, many studies have focused on the working parameters and geometry of tools.

Gustafsson et al. conducted an experimental study of the forces and energies involved in cutting steel sheets with angled tools [5]. In general, prestigious specialists in the field refer to shear-cutting tools [6] and they have shown that monitoring working conditions is essential for determining their quality, wear, and tool life. Also, Wang et al. [3] showed that high shearing force is required under severe conditions in the material shearing process, indicating that parameter optimization is necessary to improve the cutting performance.

Kolhatkar et al. provided an overview of the factors influencing cut-edge quality and tool life in table shear processes, offering insights into tool design and other influencing parameters [7].

**Figure 1:**
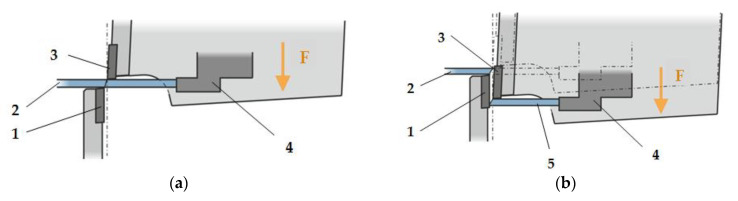
Principles of shear cutting: (**a**) before shearing; (**b**) after shearing. The elements are a (1) fixed tool; (2) cutting board; (3) movable tool; (4) limiter; and (5) cut board [9].

Mechanical shear cutting, mainly used for cutting sheets, is carried out on shear-type machines that use two blade-type tools (one fixed and the other movable); these tools are driven by different systems, which shear the blank under the action of a pressing force, F (Figure 1), [5,8].

Mechanical flow tools are made from high-quality alloy steel, capable of withstanding the complex stresses encountered during service. They must have the following operating characteristics: higher hardness than the metal material being machined; resistance to bending, compression, and tensile strength; resistance to small plastic deformations to maintain the tool’s geometry during machining; and toughness [9].

**Figure 2:**
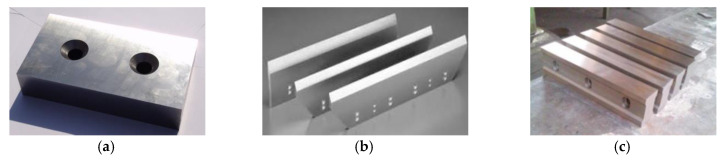
Constructive types of shearing tools: (**a**) straight; (**b**) inclined; and (**c**) profiled.

Shear tools (Figure 2) are available in a wide range of shapes and sizes, depending on the shears on which they are mounted. They are usually made of Cr-V and W alloyed steels, which are materials capable of withstanding complex stresses due to the technological cutting process as well as the wear and tear from the cutting operation.

**Figure 3:**
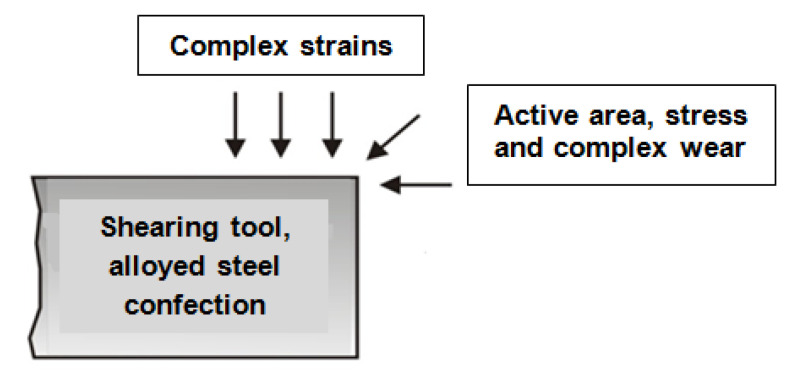
Areas of complex stresses and wear on shearing tools.

During operation, the active surfaces of the tools are exposed to complex wear: abrasive wear under medium and high pressure, adhesion wear, corrosion, and fatigue [10]. In Figure 3, the areas where the complex stresses occur and the types of wear that are predominantly manifested on the active edges are schematized.

Over time, their active edges are subjected to complex wear and tear and deteriorate, requiring them to be re-sealed at various intervals, after which they are removed and replaced with new ones.

Considering the European Commission Directives [1], which aim to significantly reduce consumption and protect the environment, attention has been directed toward developing reliable tools by using new concepts and technologies, enabling the reuse, modernization, and repair of tools.

In the field of product repair, modern technologies have been developed that focus on the reconditioning of products through weld deposition processes. These processes produce deposits of material with superior hardness to compensate for wear and tear during service. The application of these processes not only makes it possible to recover products with defects, such as cracks or partial ruptures caused by wear and tear, but also increases their durability and, consequently, their reliability in service [11].

Research in the field of welding reconditioning has been carried out on tools such as dies for plastic deformation [12,13], cutting tools [10], hammers for the mining industry [14], and automotive components [15,16].

Filler materials play an important role in electric arc fusion welding deposition processes. Thus, specialists in the field [17] have demonstrated that the addition of titanium to the powder in tubular wires positively affects the wear resistance of the deposited layers. In [18], the effects of different additive materials on the weld repair of tools used for hot plastic deformation were examined.

Based on these premises, the paper presented innovative technology for manufacturing shear tools, consisting of a support structure in which interchangeable modular elements are mounted. The support structure is made of strong, non-alloy, low-cost, non-alloy steel that is capable of withstanding complex operating stresses. The modular elements, also made of non-alloy steel, are deposited into active areas (surfaces) with welded layers (cords) that include alloyed additives to withstand operating stresses. They offer the possibility of periodic machining at operating speeds according to wear and allow for rapid interchangeability. In this respect, the scope of the paper was not to optimize a manufacturing process, but the purpose was to implement new technology for shear tool manufacturing.

Manufacturing through this process increases the durability and reliability of the tools while greatly reducing manufacturing, operating, and maintenance costs.

## 2. Materials and Methods

### 2.1. Manufacturing Shearing Tools from Interchangeable Plates Made by Depositing Cords in Active Areas

**Figure 4:**
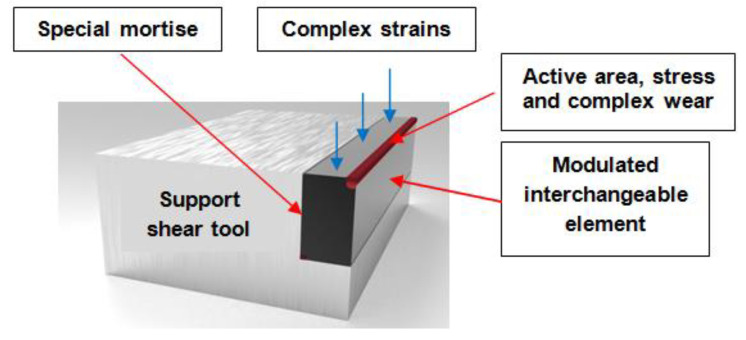
The principle of placing modulated elements.

This paper presents modern technology for manufacturing cutting tools, consisting of a supporting structure that has the shape and dimensions of the cutting tool and includes a special recess in which the interchangeable modular element is mounted. The supporting structure is made of tough, non-alloy, low-cost, non-alloy steel that is capable of withstanding the complex stresses of the operation. The modulated elements, also made of non-alloy steel, are loaded in active areas (surfaces) with welded layers (cords) that include alloyed additives to withstand operating stresses. This allows for periodic machining at operational rates, depending on wear, as well as rapid interchangeability (Figure 4).

**Figure 5:**
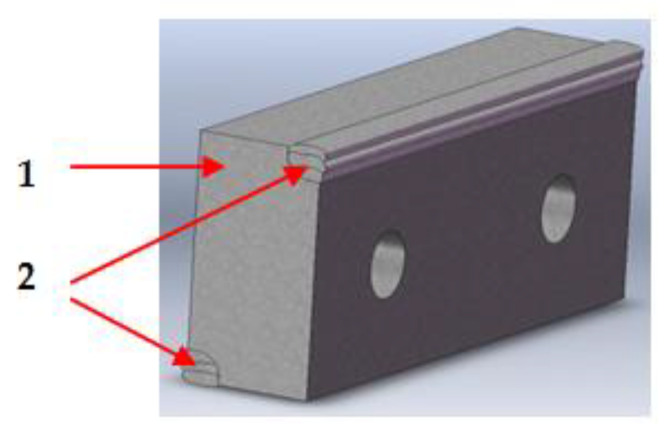
Interchangeable modulated elements manufactured by deposition: (1) plate; (2) active edges loaded with welding cords.

The interchangeable modulated elements are in the form of parallelepiped plates, also made of carbon or low-alloy steel, which are welded at the active edges with alloyed filler materials that are resistant to the stresses in service (Figure 5).

**Figure 6:**
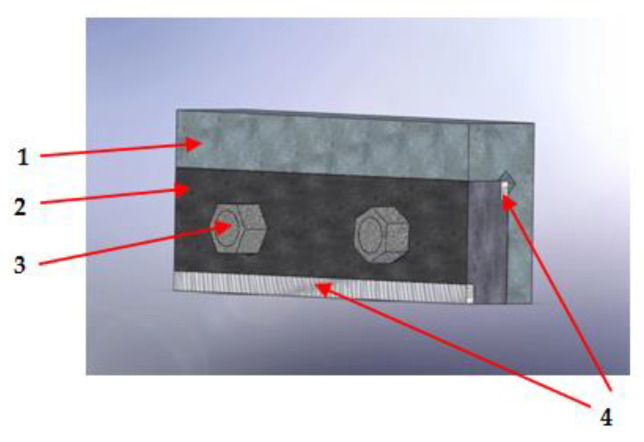
Shear tool with interchangeable modulated elements: (1) tool holder; (2) interchangeable modulated element; (3) fastening element; and (4) deposit (cord).

The assembly of the modulated elements in the knife holder was carried out using bolt-type fasteners, inserted into specially designed holes (Figure 6).

The first active edge, loaded by welding, was used until the modulated element wore out, after which the modulated element rotated 180°. After complete wear of the second edge, the interchangeable element was replaced.

The study used a sample of six shearing tools for cutting boards up to 10 mm thick. They were made of interchangeable modular elements, according to the principles presented above, and were mounted on specific types of shears.

**Figure 7:**
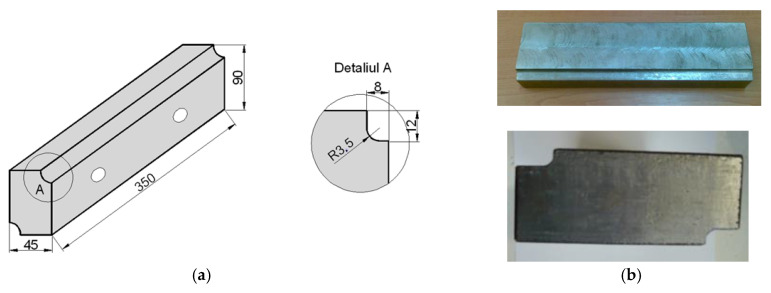
Shapes and dimensions of plates for interchangeable modular elements: (**a**) machining dimensions; (**b**) appearance of the machined plate in active areas for deposition.

The plates for the tested interchangeable modulated elements were made according to Figure 7 and adapted to the dimensions of the sharing tool in steel type S 355 JR. The choice of material was based on its high toughness, thus constituting the basic structure of the tool capable of withstanding complex operational stresses (compression, bending, etc.), except for wear that is taken up by the deposited surfaces of the modulated element. These surfaces have been machined by mechanical processes at the opposing active edges to provide a base for the deposition of the weld beads.

The welding deposition was carried out using the manual electric arc welding process with a covered electrode, specifically designed and manufactured for this purpose. The electrode type is made by EICr2.5W4.5V La [10,12] and is capable of achieving deposits with medium hardness.

A coated electrode was designed for deposition by electric arc welding, consisting of an iron base, with additions of chromium, tungsten, vanadium, and lanthanides. The material was developed in the form of a coated electrode, that is, an unalloyed metal rod with a coating containing alloying elements and lanthanides. The beads deposited by electric arc welding created an iron-based alloy with the following alloying elements: carbon 0.2–0.5%, silicon 1–1.5%, chromium 2–3%, vanadium 0.4–1%, and tungsten 3–5.5%, denoted as EiCr2.5W4.5 V La [10,12]. The alloy had a maximum hardness of 45HRC (in the welded state).

**Figure 8:**
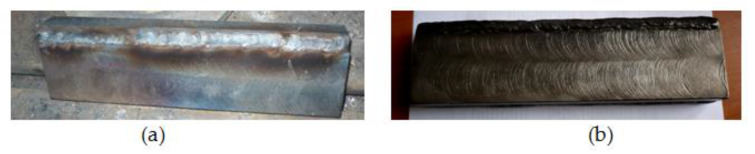
Deposit aspect: (**a**) rot bead; (**b**) surface bead.

Three weld beads were deposited on each edge using the parameters shown in Table 1. Additionally, the deposit aspects are shown in Figure 8.

### 2.2. Lifetime Statistical Analysis of Different Types of Shearing Tools

A lifetime analysis consists of an inferential study of the main estimated distribution parameters [19,20,21] for different types of shear tools manufactured through various deposition welding.

The analyzed shearing tools were as follows:

- Type I: shear tool manufactured by a conventional process;
- Type II: shear tool manufactured with interchangeable modular elements loaded by deposition welding with a covered electrode (Figure 8).

The six tested tools were mounted on specific types of shearing; operating times were recorded until complete wear of each active edge. Wear control was visually performed.

The type II shear tool had two active edges loaded by welding. After the first edge wore out, the interchangeable modular element was rotated 180° and the second edge was used until completely worn out.

The lifetime estimation for analyzed shearing tools was made using the Minitab 17 software (Minitab LLC, State College, PA, USA).

**Figure 9:**
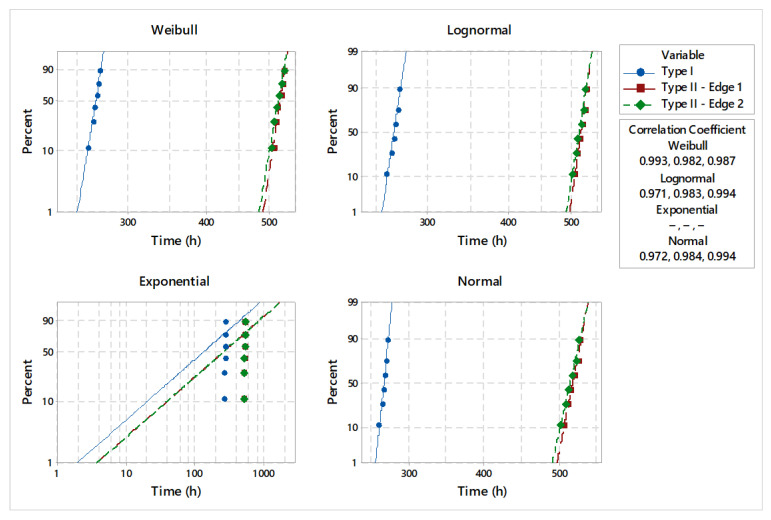
Statistical model validation.

Considering four common distributions in the field of product reliability estimation, the statistical model validation was made by applying the goodness-of-fit test [20]. The results of the distribution analysis are presented in Figure 9. Comparative analysis of the goodness-of-fit test shows that lifetime data did not fit the supposed distribution for low statistic values (Table 2, Table 3 and Table 4). Comparing the values of the correlation coefficients, it can be seen that they were comparable, except for the exponential distribution. Specific to the type I shear tool, the goodness-of-fit test presents very good results, highlighted by correlation coefficient values of 0.97 and 0.99. For both edges of the analyzed type II shearing tools, the goodness-of-fit test indicates a very good correlation with the analyzed data. Additionally, based on the adjusted Anderson–Darling (AD*) statistics and the estimated correlation coefficients of 0.98 and 0.99, it can be suggested that the analyzed data follow the Weibull distribution.

The estimated mean time to failure (MTTF) indicates a minimum value of 266 h for the type I shearing tool and a maximum value of 518 h for the type II—edge 1 shearing tool (Table 5).

Specifically for the Weibull distribution, and applying the least squares method (LSM) [21] with a 95% confidence interval (CI), the parameters for two types of shearing tools were estimated. The distribution analysis consisted of a parametric estimation of the main parameters for the type I and type II shearing tools.

The statistical characteristics of the estimated distribution parameters are detailed in Table 6 and Table 7. The estimated shape parameters highlight comparable values for operating times, with differences considered insignificant. A more detailed investigation of the main predicted statistical parameters shows that the type II shearing tool has double the lifetime on each edge compared to the type I shearing tool. With a mean time to failure of 518 h, a median of 519 h, and a standard deviation of around 9 h, the type II—edge 1 shearing tool demonstrates superior lifetime performance during the operating time. In this case, the estimated first quartile (Q1) is 513.14 h, the third quartile (Q3) is less than or equivalent to 524.75 h, and the computed interquartile range (IQR) is around 11 h. In contrast, the type I shearing tools have a first quartile (Q1) value of 263.22 h, a Q3 (third quartile) that is smaller than or equivalent to 269.76 min, and an estimated interquartile range of approximately 6.53 operating hours.

The main statistical parameters of the analyzed shearing tool types are synthetically presented in Table 6.

**Figure 10:**
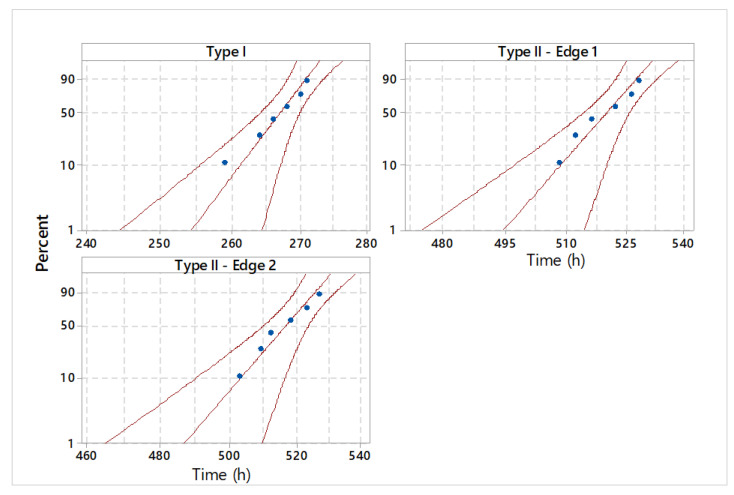
Probability plot for analyzed shear tools.

**Figure 11:**
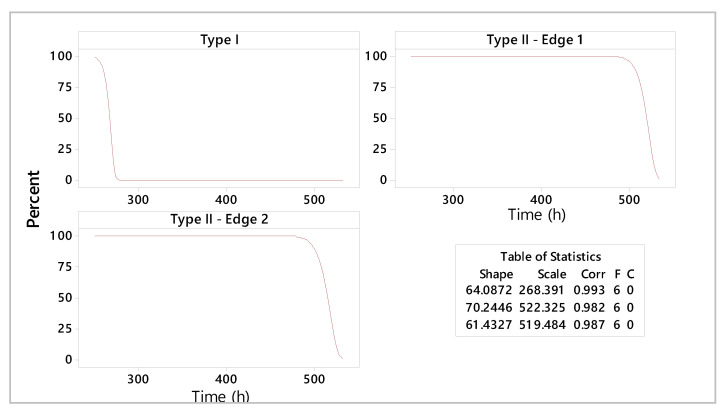
Reliability functions of analyzed shear tools.

**Figure 12:**
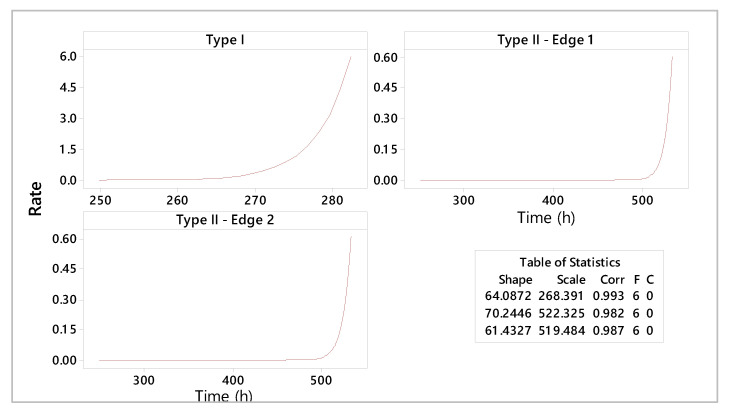
Hazard plot of analyzed shear tools.

The graphical statistical analysis is highlighted by probability plots (Figure 10), reliability functions (Figure 11), and hazard rate plots (Figure 12).

As a result of the estimated parameters, it can be stated that type II shearing tools have higher lifetimes compared to type I shearing tools. The increased reliability of these tools is indicated by scale parameters with estimated values of 522 h and 519 h of operation in exploitation, respectively. Specific failure rate graphs (Figure 12) show an increase in wear for type I shearing tools after 270 h, while type II shearing tools indicate a deterioration phenomenon accentuated after approximately 520 h of use.

## 3. Results

### 3.1. Analysis of Interchangeable Modular Elements

The following determinations were carried out on the interchangeable modular elements, which were made by weld deposition on both active edges, and machined to the shape and dimensions of use, namely, the chemical composition of the deposited metal, macro- and microstructural analyses, and hardness measurements.

- Chemical composition of the deposited metal:

The chemical compositions of the deposition metal were determined using a Spetromax optical emission spectrometer, as presented in Table 8.

**Figure 13:**
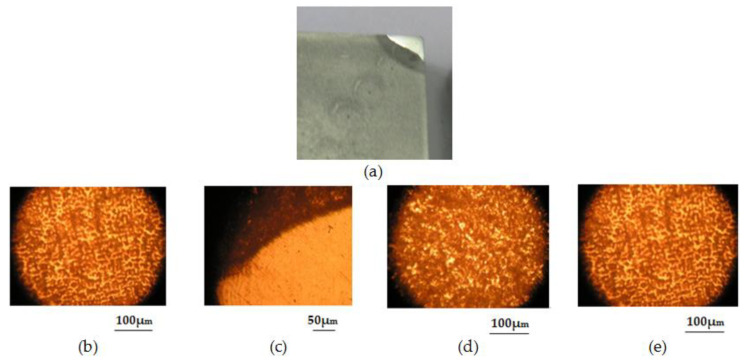
Macrostructural and microstructural analysis images of the deposited area: (**a**) plate macrostructure; (**b**) deposited material (DM), 100× magnification—martensitic casting structure with retained austenite and uniformly distributed complex carbides; (**c**) transition area (TA), 50× magnification—complete melting without defects; (**d**) heat-affected area (HAZ), 100× magnification—martensite and bainite; (**e**) base material (BM), 100× magnification—pearlitic–ferritic structure.

- Macro- and microstructural analyses:

On a cross-section in the loaded zone, macro- and microstructural determinations were performed. The zones specific to weld deposition base metal (BM), deposition zone (DM), transition zone (TA), and heat-influenced zone (HAZ), respectively, were analyzed (Figure 13). The preparation of metallographic samples for macro- and microscopic analyses was conducted in accordance with EN 1321:2000. The microstructural analyses were carried out on a Nikon (metallographic) optical microscope, the Eclipse MA 100 model.

**Figure 14:**
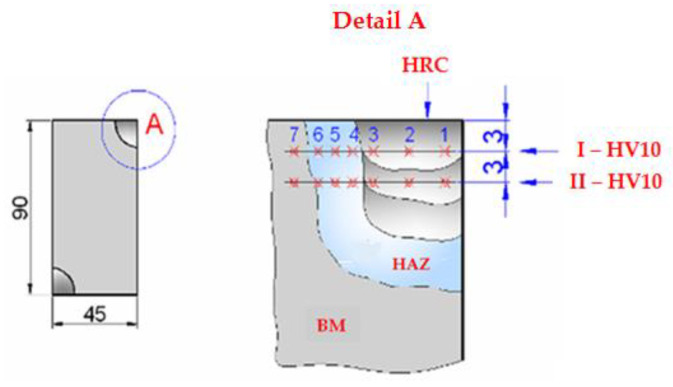
The cross-section in the modulated element made by the deposition of weld cords in the active zones. Points 1–7 indicate where the microhardness (HV 10) was measured.

- Hardness measurements:

The microhardness values (HV10) in specific areas of the deposition welding (Figure 14), are shown in Table 9; they show little variation in the values recorded for each zone.

The microhardness (HV) measurements were carried out in cross-sections in two directions within the specific deposition zones (DM, HAZ, BM), as well as HRC hardness measurements on the active outer surface after machining to the operating dimensions.

The HRC hardness measurements (Figure 14) were carried out within the active outer surface of the cords deposited on the modulated element, along their entire length at 20 mm intervals. The values obtained are recorded in Table 10.

The hardness measurements (in deposition areas) were taken using an FM 700 microdurometer.

The analysis of the performed measurements, presented in Table 9 and Table 10, shows that the hardness values of the metal deposited by weld on active areas (edges) of the modulated elements were in accordance with the product standards.

### 3.2. Comparative Lifetime Analysis of Shearing Tools

**Figure 15:**
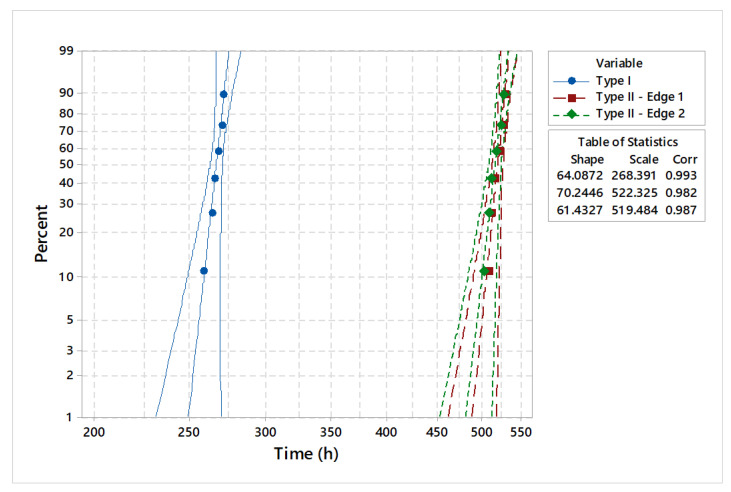
Comparative analysis of the probability plot.

An overall comparative distribution analysis is shown in Figure 15. With 70.24 h of shape parameter estimation, the type II—edge 1 shearing tool indicates the best lifetime period of 522.32 h. At the opposite end is the type I shearing tool, with 268.39 h of operating time.

**Figure 16:**
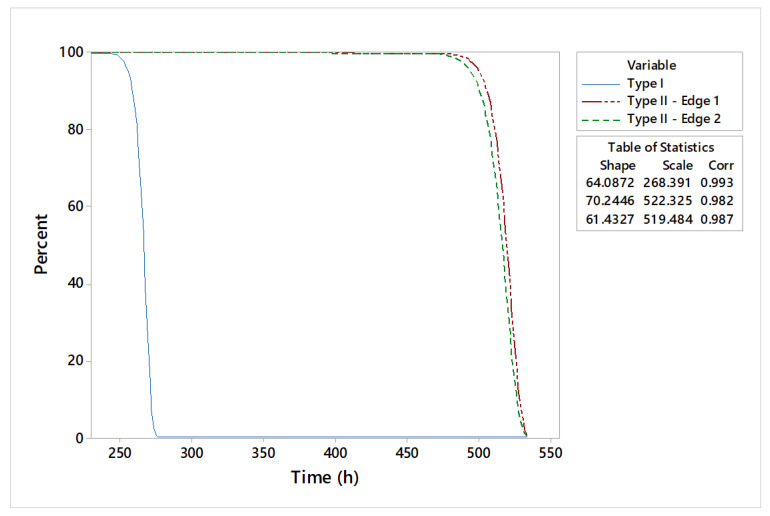
Lifetime predictions for different constructive shear-cutting tools.

**Figure 17:**
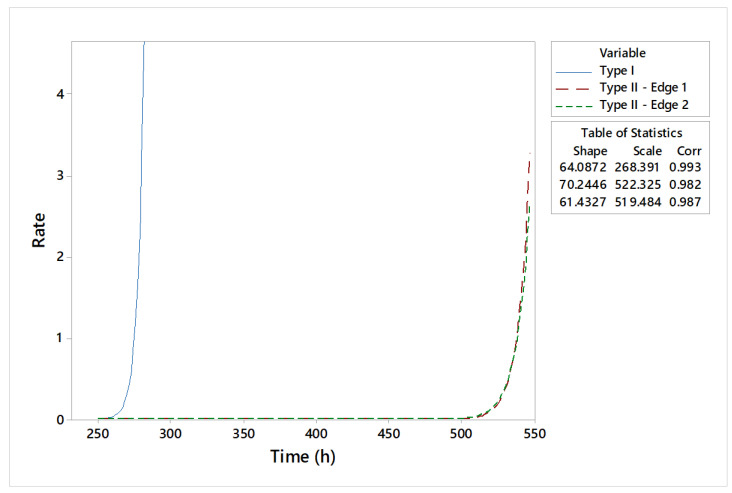
Comparative analysis: hazard plot.

The tool reliability analysis consists of lifetime prediction for different constructive shear-cutting tools (Figure 16 and Figure 17).

Interpretations of statistical and experimental results highlight the high reliability of type II shearing tools. A detailed analysis of the estimated reliability parameters shows that the maximum period of use for the type I shearing tool is 268 h, for type II—edge 1, it is 528 h, and for type II—edge 1 it is 526 h. Moreover, the shapes of the specific curves of reliability functions highlight an accentuated phenomenon of wear. The hazard rate trend for the type II shearing tool shows smooth variations over the operating time compared to the analyzed type I shearing tool.

## 4. Discussion

The justification for an individual analysis of the two types of tools was based on the shearing tool failure analysis and the complex stresses to which they are subjected during operation. These factors can lead to damage to the active edges and, implicitly, to a reduction in the tool’s lifetime.

Traditional shear tools experience significant wear due to complex operational stresses, necessitating frequent replacements and increasing maintenance costs. By integrating modular elements with alloyed welded deposits on active cutting edges, the proposed design improves the tool’s lifetime while reducing material waste and production costs. In order to achieve the conception and realization of this purpose-built electrode, other brands of electrodes were also tested within the framework of the studies conducted by authors [10,12].

In cutting deformation processes, the shearing angle is a critical factor. Numerical and experimental investigations of shearing tool characteristics were developed in [22]. Under high-speed cutting conditions, different shearing angle models were developed and focused on through computational simulation models for shearing of shearing angle and chip thickness. The experimental and simulation results show that the shear angle variations are influenced by cutting speed, feed rate, friction behavior, and material properties.

In the field of sheet metal shearing, optimizing shearing tool profiles [7] is crucial for ensuring an efficient process with minimal tool wear and superior edge quality. Analyzing the geometric and functional parameters of the blade enables improvements in the overall cutting performance.

The essential characteristics of shearing tool profiles are as follows: tool geometry and shape [22,23], cutting angle and tip radius [24], tool material and surface treatments, and the anisotropy of the cutting material.

Regarding the technical aspects and performance parameters, the cutting force is influenced by multiple factors, including blade geometry, cutting speed, material properties, and contact parameters between the shearing tool and the workpiece. An optimized profile can reduce mechanical stress and contribute to lower energy consumption during the operation.

One of the primary indicators of cutting efficiency is the burnish length obtained on the sheared edge. A smoother surface with a reduced fracture zone indicates better control of plastic deformation and material separation. In this context, adjusting blade geometry and optimizing cutting parameters are essential.

The analysis of different types of shearing tools could help to optimize the cutting process. Compared to shear tools manufactured by conventional processes, the results highlight a considerable increase in reliability—specifically in terms of lifetime—for shear tools manufactured with interchangeable modular elements loaded by deposition welding using covered electrodes.

Optimizing the profile of cutting tools involves a multidisciplinary approach that combines geometric analysis, material selection, and process parameter adjustment. By implementing innovative profile designs and advanced surface treatments, significant reductions in cutting forces, improved edge quality, and increased tool durability can be achieved. These factors are essential for enhancing processing efficiency and reducing operational costs in industries that rely on metal shearing processes. The distance between the cutting blade and the support plays a crucial role in achieving precise cuts. An optimized clearance prevents excessive burr formation and minimizes tool stress. Experimental studies have shown that a clearance that is too small can lead to premature tool wear, while a clearance that is too large can affect edge quality.

During the shearing process, heat accumulation can accelerate tool wear and affect operation precision. The use of geometry optimized for thermal dissipation, combined with high thermal conductivity materials, can help to reduce the temperatures in the shearing zone. Studies have shown that optimized blade profiles can lower contact temperatures, positively impacting tool durability [5,10,22,23,24].

The importance of the appropriate design and manufacturing of shearing tools is substantiated by the increased operational duration under specified conditions. In this regard, studying the reliability and failure rate of shearing tools plays an essential role. The analysis and interpretation of experimental results enabled the identification of constructive technical solutions, as well as the implementation of adequate maintenance strategies and decisions.

The main advantages of shearing tools include the following:

- Use of cheap manufacturing materials.
- Flexible modular interchangeable fixing system.
- High productivity.
- Long operating lifetime, as proven by the study on the reliability estimation of shearing tools, taking into account the two active edges.
- Reduced manufacturing costs due to weld deposition, with loading carried out only in the area of the active edges.
- The process used does not require additional training or qualifications for operators.

## 5. Conclusions

This study proposes an economical alternative for manufacturing shearing tools, using inexpensive materials and loaded only in the active edge area by welding processes. The materials used for fabrication are affordable and do not require expensive equipment for the welding loading process.

This paper focuses on enhancing the durability and reliability of shearing tools through the integration of interchangeable modular elements through welding deposition technology. The researches highlights the advantages of this modular design over conventional shearing tools, demonstrating significant improvements in both lifetime and wear resistance.

The adopted technological solution allows for processing at operating levels, depending on the wear phenomenon, as well as rapid interchangeability. These shearing tools with modular elements are made according to the dimensions of the supports on which they are mounted, taking into account the technical documentation developed for each type. After welding deposition, their final shape is achieved through classical processes. The reliability analysis, using Weibull distribution modeling, confirms that the proposed modular tool design more than doubles the operational lifespan compared to traditional shear tools. The mean time to failure (MTTF) for the modular design exceeds 500 h per cutting edge, compared to approximately 266 h for conventionally manufactured tools. This extended lifetime is attributed to the robust alloyed welded deposits on the active edges, which provide superior resistance to mechanical and abrasive wear.

Experimental results validate the effectiveness of the proposed method, showcasing the improved performance and sustainability of the shearing tools. The modular design allows for easy replacement of worn elements, reducing material waste and maintenance costs while maintaining operational efficiency.

Furthermore, the study emphasizes the economic and environmental benefits of this approach. The use of affordable materials, combined with efficient manufacturing techniques, supports sustainable production practices. The ability to replace individual modular elements rather than entire tools reduces resource consumption and aligns with the European Commission directives on sustainability and material efficiency.

In summary, the findings suggest that the adoption of modular shearing tools with weld deposition technology offers a viable and cost-effective solution for industrial applications. The research provides valuable insights for further exploration into optimizing tool design, enhancing material properties, and implementing predictive maintenance strategies to maximize operational efficiency and reliability in manufacturing environments.

## Figures and Tables

**Table 1:** Technical welding parameters.

Electrode Mark	Diameter (mm)	Welding Parameters			
		Welding Current (A)	Nat. Welding Current	Arc Tension (V)	Welding Speed (m/min)
EiCr2.5W4.5 V La	3.25	120 ± 10	CC+	20–24	0.15–0.2

**Table 2:** Goodness-of-fit for type I shear tools.

Distribution	Anderson–Darling (AD*)	Correlation Coefficient
Weibull	1.99	0.99
Lognormal	2.07	0.97
Exponential	5.73	–
Normal	2.06	0.97

**Table 3:** Goodness-of-fit for type II shear tools—edge 1.

Distribution	Anderson–Darling (AD*)	Correlation Coefficient
Weibull	2.03	0.98
Lognormal	2.02	0.98
Exponential	5.73	–
Normal	2.02	0.98

**Table 4:** Goodness-of-fit for type II shear tools—edge 2.

Distribution	Anderson–Darling (AD*)	Correlation Coefficient
Weibull	2.02	0.98
Lognormal	1.98	0.99
Exponential	5.71	–
Normal	1.98	0.99

**Table 5:** Results of mean time to failure (MTTF).

Distribution	MTTF		
	Type I (h)	Type II—Edge 1 (h)	Type II—Edge 2 (h)
Weibull	266.03	518.13	514.73
Lognormal	266.34	518.69	515.36
Exponential	185.21	360.62	358.91
Normal	266.33	518.66	515.33

**Table 6:** Weibull parameter estimation.

Shear Tool Type	Parameter	Estimate (h)
Type I	Shape	64.08
	Scale	268.39
Type II—Edge 1	Shape	70.24
	Scale	522.32
Type II—Edge 2	Shape	61.43
	Scale	519.48

**Table 7:** Distribution characteristics.

Parameter	Type I (h)	Type II—Edge 1 (h)	Type II—Edge 2 (h)
Mean (MTTF)	266.03	518.13	514.73
Standard deviation	5.26	9.36	10.62
Median	266.86	519.60	516.39
First quartile (Q1)	263.22	513.14	509.05
Third quartile (Q3)	269.76	524.75	522.25
Interquartile range (IQR)	6.53	11.61	13.19

**Table 8:** Chemical compositions of the deposition metal [12].

Chemical Compositions (%)								
C	Mn	Si	Cr	Mo	Ni	V	W	Co
0.2–0.5	-	1–1.5	2–3	max 1	max 1.5	0.4–1	3–5.5	-

**Table 9:** The microhardness values (HV 10) measured in cross-sections within the specific deposition zones.

Direction/Specific Areas	DM			HAZ			BM
	1	2	3	4	5	6	7
HV 10 Direction I (Weld bead 1)	736	690	752	252	248	249	192
HV 10 Direction II (Weld bead 2)	530	565	572	230	237	228	195

**Table 10:** The HRC hardness values measured on the active surface of the deposited strands.

Specific Areas	Cords Surface									
	1	2	3	4	5	6	7	8	9	10
HRC	53	56	52	54	53	55	56	53	54	52

## Data Availability

The original contributions presented in this study are included in the article. Further inquiries can be directed to the corresponding author.

## References

[1] European Union Sustainable Development Goals—European Commission. https://international-partnerships.ec.europa.eu/policies/sustainable-development-goals_en.

[2] Dubey A.K., Yadava V. (2008). Laser beam machining—A review. Int. J. Mach. Tools Manuf..

[3] Wang H., Ma Y., Bai Z., Liu J., Huo L., Wang Q. (2022). Experimental investigation and finite element modeling for improved shearing cutting performance using optimized bio-inspired shearing tool. J. Braz. Soc. Mech. Sci. Eng..

[4] Colombo V., Concetti A., Ghedini E., Dallavalle S., Vancini M. (2009). High-speed imaging in plasma arc cutting: A review and new developments. Plasma Sources Sci. Technol..

[5] Gustafsson E., Karlsson L., Oldenburg M. (2016). Experimental study of forces and energies during shearing of steel sheet with angled tools. Int. J. Mech. Mater. Eng..

[6] Lara de Leon M.A., Kolarik J., Byrtus R., Koziorek J., Zmij P., Martinek R. (2024). Tool condition monitoring methods applicable in the metalworking process. Arch. Comput. Methods Eng..

[7] Kolhatkar A., Pandey A. (2023). Sheet Metal Shearing Process: An Overview. Trans. Indian Natl. Acad. Eng..

[8] RAS Comparison: Swing Beam Shears—Guillotine Shears. https://www.ras-systems.com/products/cutting/comparison-swing-beam-shears-guillotine-shears.

[9] Amza G. (2002). Tratat de Tehnologia Materialelor.

[10] Iovanas D.M. (2007). Researches Regarding the Technology and Reliability of Technological Equipments Reconditioned by Welding. Ph.D. Thesis.

[11] Binchiciu H., Iovanas R. (1992). Loading by Welding with Electric Arc.

[12] Iovanas D.M., Dumitrascu A.E. (2024). Lifetime analysis of dies manufactured by conventional processes and reconditioned by deposition welding operation. Materials.

[13] Machedon Pisu T., Vas A., Magyari M., Iordache A. (2013). Research on cladding (CMT MIG, WIG ARC mechanized pulse) for molds used for casting. Metal. Int..

[14] Saceanu C., Rosu R.A., Pascu D.R. (2012). Endurance evaluation of the deposited layers on the coal hammers mills. Metal. Int..

[15] Chivu O., Rontescu C., Cicic D.T., Balan G. (2016). The effects of reconditioning by welding of crankshafts in automotive industry. Metalurgija.

[16] Chivu O., Cicic D.T., Vasile I.M. (2016). The influence of the reconditioning by welding processes on the hardness of crankshafts in the automotive industry. Metalurgija.

[17] Shlyarov V.V., Komarov A.A., Kozyrev N.A., Polevoi E.V., Mikhno A.R. (2023). Microstructure and Tribological properties of metal layer deposited by arc cladding of powder wire containing titanium powder. Met. Sci. Heat. Treat..

[18] Szovák B., Korsós K., Kemény D.M., Szalva P. (2023). Effect of the Welding Filler Material on the Mechanical and Corrosive Behavior of Bohler W350 ISOBLOC Hot Forming Tool Steel. Period. Polytech. Mech. Eng..

[19] Jiang R. (2015). Introduction to Quality and Reliability Engineering.

[20] Dai W., Sun J., Chi Y., Lu Z., Xu D., Jiang N. (2019). Review of Machining Equipment Reliability Analysis Methods based on Condition Monitoring Technology. Appl. Sci..

[21] Gourdin E., Hansen P., Jaumard B. (1994). Finding Maximum Likelihood Estimators for the Three-Parameter Weibull Distribution. J. Glob. Optim..

[22] Hao M., Xu D., Feng P. (2019). Numerical and experimental investigation of the shear angle in high-speed cutting of Al6061-T6. Int. J. Adv. Manuf. Technol..

[23] Sutter G. (2005). Chip geometries during high-speed machining for orthogonal cutting conditions. Int. J. Mach. Tool. Manuf..

[24] Tamizharasan T., Kumar N.S. (2012). Optimization of cutting insert geometry using Deform-3D: Numerical simulation and experimental validation. Int. J. Simul. Model..

